# Myocardin‐related transcription factor A drives ROS‐fueled expansion of hepatic stellate cells by regulating p38‐MAPK signalling

**DOI:** 10.1002/ctm2.688

**Published:** 2022-02-20

**Authors:** Wenhui Dong, Ming Kong, Hong Liu, Yujia Xue, Zilong Li, Yutong Wang, Yong Xu

**Affiliations:** ^1^ Key Laboratory of Targeted Intervention of Cardiovascular Disease and Collaborative Innovation Center for Cardiovascular Translational Medicine, Department of Pathophysiology Nanjing Medical University Nanjing China; ^2^ State Key Laboratory of Natural Medicines, Department of Pharmacology China Pharmaceutical University Nanjing China; ^3^ Institute of Biomedical Research Liaocheng University Liaocheng China; ^4^ Department of Cell Biology, Municipal Laboratory for Liver Protection and Regulation of Regeneration, School of Basic Medical Sciences Capital Medical University Beijing China

Dear editor,

We describe in this letter a novel retrograde signalling mechanism that highlights a myofibroblast‐specific role for myocardin‐related transcription factor A (MRTF‐A), also known as MKL1, in liver fibrosis.

Liver fibrosis is considered a common pathological process in virtually all end‐stage liver diseases. Absent from the liver under physiological conditions, myofibroblasts quickly emerge and expand as a result of liver injuries to mediate the pro‐fibrogenic response. Hepatic stellate cells (HSCs), tucked between the liver parenchyma and the hepatic sinusoid, is considered as the predominant source from which myofibroblasts are derived regardless of aetiology.^1^ Reactive oxygen species (ROS) fuels HSC proliferation via a network of signalling cascades. Mitogen‐activated protein kinases (MAPKs) are well‐documented for their regulatory roles in ROS‐driven HSC proliferation and liver fibrosis by programming transcriptional events in the nucleus. How the cytoplasm‐nucleus crosstalk influences MAPK signalling and contributes to ROS‐dependent HSC proliferation is not well understood. MRTF‐A is a transcriptional modulator implicated in the pathogenesis of a wide range of human diseases. Despite the near‐unanimous view that MRTF‐A is a pivotal regulator of myofibroblast trans‐differentiation,^2^ there is no direct genetic evidence to support or refute the hypothesis that myofibroblast‐specific MRTF‐A deletion is sufficient to suppress liver fibrosis in vivo due to its universal expression pattern.^3^

We first evaluated the possibility that the ability of MRTF‐A to regulate liver fibrosis might be myofibroblast‐autonomous. *MRTF‐A* ^f/f^ mice were crossed to *Postn*‐Cre^ERT2^ mice to generate myofibroblast‐conditional MRTF‐A deletion mice (*MRTF‐A* ^ΔMF^) followed by carbon tetrachloride (CCl_4_) injection to induce liver fibrosis. Western blotting indicated that MRTF‐A expression was markedly decreased in HSCs, but not in hepatocytes, from *MRTF‐A* ^ΔMF^ mice, compared to *MRTF‐A* ^f/f^ mice following tamoxifen injection (Figure S1). As shown in Figure 1A,B, plasma alanine aminotransferase/aspartate aminotransferase (ALT/AST) levels were comparable between the *MRTF‐A* ^f/f^ mice and the *MRTF‐A* ^ΔMF^ mice. Quantitative polymerase chain reaction (qPCR) analysis (Figure 1C), picrosirus red/Masson's trichrome staining (Figure 1D) and hepatic hydroxylproline quantification (Figure 1E) all supported the notion that myofibroblast‐specific MRTF‐A deficiency is sufficient to dampen liver fibrosis. MRTF‐A deletion in myofibroblasts rendered them less capable of proliferating as evidenced by reduced expression levels of desmin (Figure 1F), a marker of HSCs. In addition, there were fewer dihydroethidium/alpha smooth muscle actin (DHE/α‐SMA)‐double positive cells in the *MRTF‐A* ^ΔMF^ livers than in the *MRTF‐A* ^f/f^ livers suggesting that MRTF‐A deficiency dampened ROS production in myofibroblasts (Figure 1G). Over‐expression of a constitutively active (CA) MRTF‐A in LX‐2 cells significantly enhanced ROS production (Figure 2A) and proliferation (Figure 2B). Treatment of N‐acetyl cysteine (NAC) completely blunted the pro‐oxidative and the pro‐proliferative effects of MRTF‐A (Figure 2A,B). Similar results were obtained in primary murine HSCs (Figure 2C,D). Of interest, over‐expression of MRTF‐A CA markedly enhanced p38‐MAPK phosphorylation (Figure 2E,F). On the contrary, MRTF‐A depletion dampened p38 phosphorylation (Figure 2G,H). Treatment with a specific p38 inhibitor completely abrogated the induction of ROS production and proliferation of HSCs (Figure 2I,J).

**FIGURE 1:**
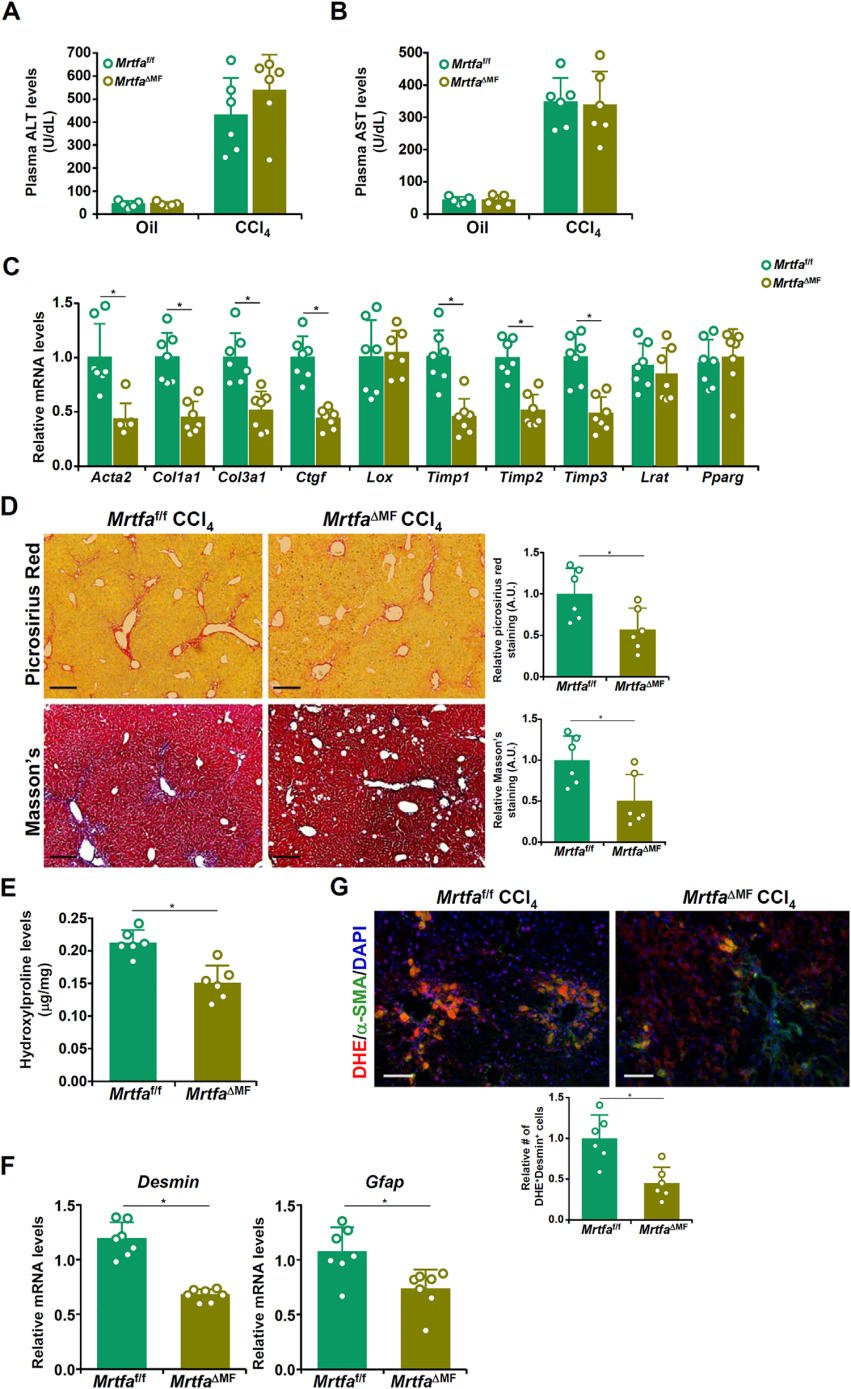
Myofibroblast‐conditional myocardin‐related transcription factor A (MRTF‐A) knockout mice display attenuated liver fibrosis. *MRTF‐A* ^ΔMF^ and *MRTF‐A* ^f/f^ mice were injected with CCl_4_ for 4 weeks as described in the Methods section. (A) Plasma ALT levels. (B) Plasma AST levels. (C) Expression of pro‐fibrogenic genes was examined by qPCR. (D) Picrosirius red and Masson's trichrome staining. (E) Hydroxylproline levels. (F) Desmin expression was examined by qPCR. (G) Frozen sections were stained with DHE and an anti‐α‐SMA antibody. *N *= 5∼6 mice for each group

**FIGURE 2:**
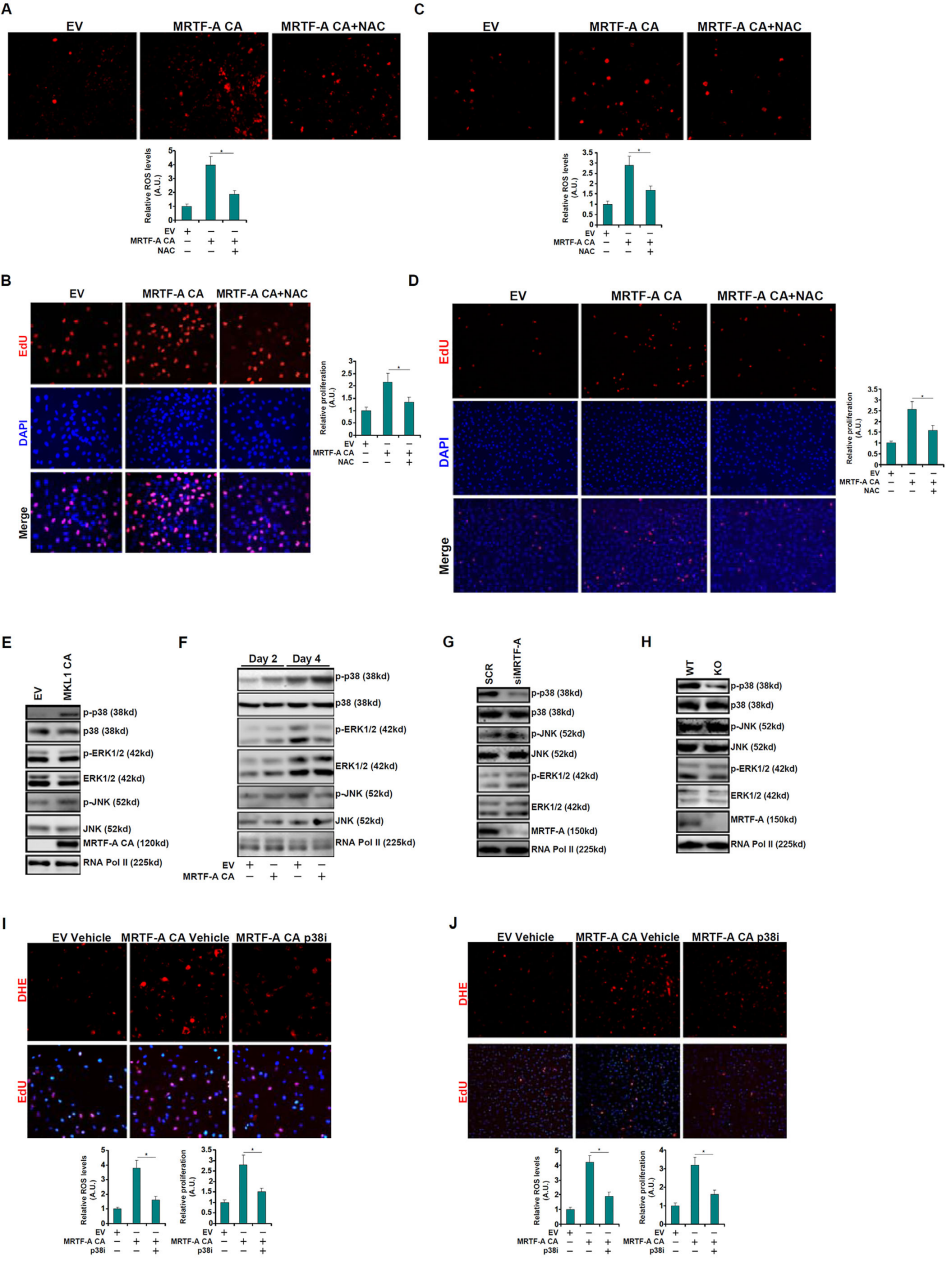
MRTF‐A promotes hepatic stellate cell (HSC) proliferation in a ROS‐dependent manner. (A, B) LX‐2 cells were infected with lentivirus carrying MRTF‐A constitutively active (CA) or an empty vector (EV) in the presence or absence of N‐acetyl cysteine (NAC; 5 mM). ROS levels were evaluated by DHE staining. Proliferation was examined by 5‐ethynyl‐2′‐deoxyuridine (EdU) staining. (C, D) Primary murine HSCs were isolated from C57/B6 mice and infected with lentivirus carrying MRTF‐A CA or an EV in the presence or absence of NAC (5 mM). ROS levels were evaluated by DHE staining. Proliferation was examined by EdU staining. (E) LX‐2 cells were infected with lentivirus carrying MRTF‐A CA or an EV followed by treatment with platelet‐derived growth factor (PDGF). Mitogen‐activated protein kinase (MAPK) phosphorylation was examined by western blotting. (F) Primary murine HSCs were isolated from C57/B6 mice and infected with lentivirus carrying MRTF‐A CA or an EV. MAPK phosphorylation was examined by western blotting. (G) LX‐2 cells were transfected with siRNA targeting MRTF‐A or scrambled siRNA followed by treatment with PDGF. MAPK phosphorylation was examined by western blotting. (H) Primary murine HSCs were isolated from wild type (WT) and MRTF‐A knckout (KO) mice. MAPK phosphorylation was examined by western blotting. (I) LX‐2 cells were infected with lentivirus carrying MRTF‐A CA or an EV followed by treatment with a p38‐MAPK inhibitor. ROS production was examined by DHE staining. Proliferation was examined by EdU staining. (J) Primary murine HSCs were infected with lentivirus carrying MRTF‐A CA or an EV followed by treatment with a p38‐MAPK inhibitor. ROS production was examined by DHE staining. Proliferation was examined by EdU staining

Based on prior discoveries that the integrin signalling pathway is intimately wired into the MAPK signalling pathway,^4^ a PCR array‐based screening was performed to identify components of the integrin pathway that can potentially be regulated by MRTF‐A. Using 2X‐fold change as a cutoff, four genes met this criterion: *Itga6* and *Ilk* were down‐regulated, whereas *Itga11* and *Itgb11* were up‐regulated in the MRTF‐A KO HSCs, compared to the WT HSCs (Figure S2). We focused on *Itga6* and *Ilk* for the remainder of the study because MRTF‐A is primarily considered an activator of transcription. Over‐expression of MRTF‐A CA robustly augmented the mRNA and protein levels of ITGA6 and ILK in vitro (Figure 3A–D) and in vivo (Figure 3E,F). Further, MRTF‐A over‐expression stimulated the ITGA6 promoter and the ILK promoter activity (Figure 3G). Chromatin immunoprecipitation (ChIP) assay confirmed that MRTF‐A was directly associated with the proximal *ITGA6* promoter and the proximal *ILK* promoter (Figure 3H). Functionally, depletion of either Integrinα6 or ILK by siRNAs rendered the cells irresponsive to MRTF‐A over‐expression by dampening ROS production, suppressing proliferation and inhibiting p38 phosphorylation (Figure S3).

**FIGURE 3:**
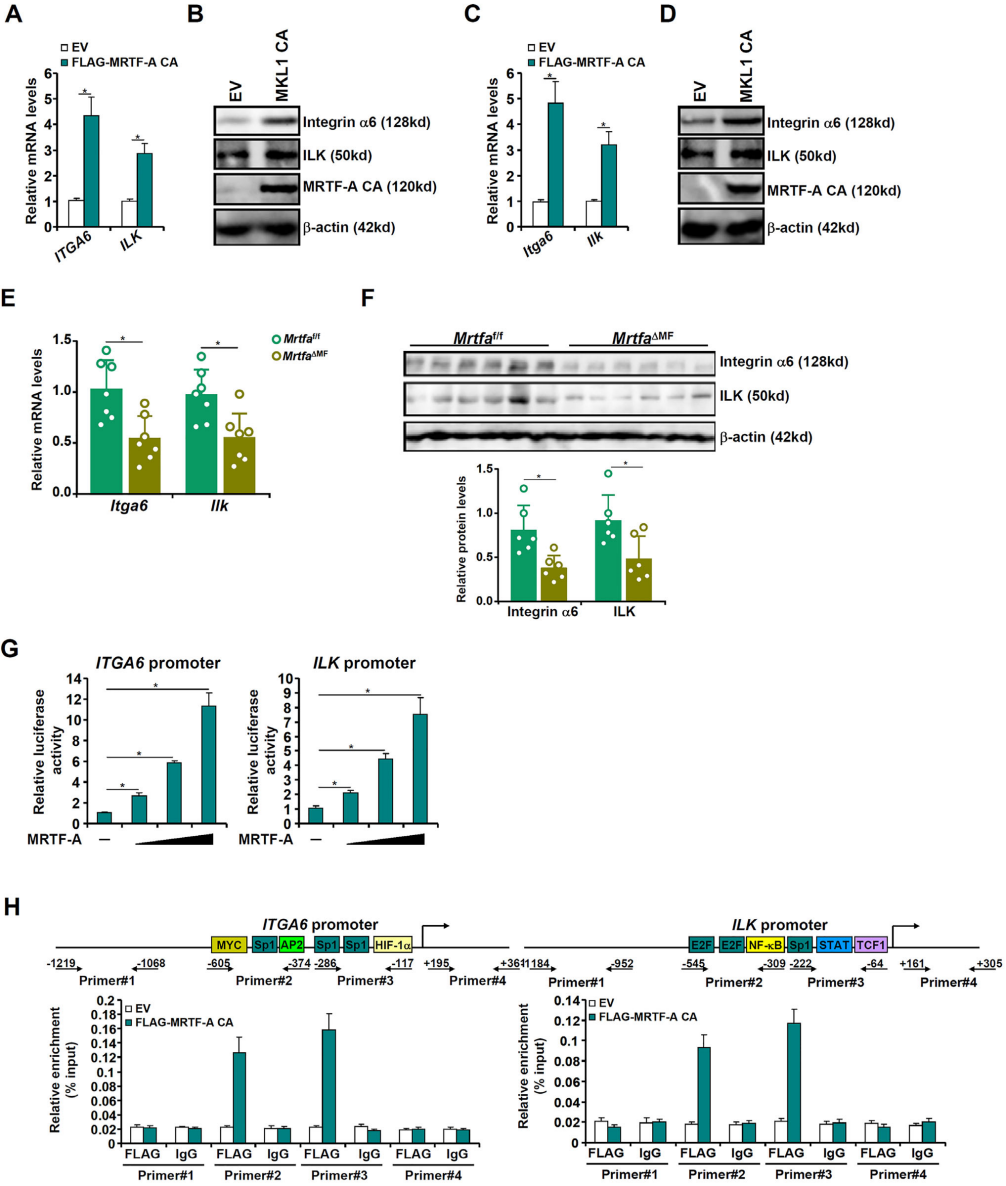
MRTF‐A regulates ITGA6/ILK transcription in HSCs. (A, B) LX‐2 cells were infected with lentivirus carrying MRTF‐A CA or an EV. *ITGA6/ILK* expression levels were examined by qPCR and western blotting. (C, D) Primary murine HSCs were isolated from C57/B6 mice and infected with lentivirus carrying MRTF‐A CA or an EV in the presence or absence of NAC (5 mM). *ITGA6/ILK* expression levels were examined by qPCR and western blotting. (E, F) *MRTF‐A* ^ΔMF^ and *MRTF‐A* ^f/f^ mice were injected with CCl_4_ for 4 weeks as described in the Methods section. *ITGA6/ILK* expression levels were examined by qPCR and western blotting. (G) LX‐2 cells were transfected with an *ITGA6* promoter construct or an *ILK* promoter construct with increasing doses of MRTF‐A. Luciferase activities were normalized by protein concentration and green fluorescence protein (GFP) fluorescence. (H) LX‐2 cells were infected with lentivirus carrying MRTF‐A CA or an EV. ChIP assays were performed with anti‐FLAG or immunoglobulin (IgG)

We finally addressed the question as to whether ILK inhibition in mice could influence liver fibrosis. The mice were injected with CCl_4_ for 4 weeks; starting at the second week, a specific ILK inhibitor (QLT‐0267) was administered peritoneally for the duration of CCl_4_ injection (Figure 4A). Plasma ALT (Figure 4B) and AST (Figure 4C) levels indicated that liver injury was ameliorated as a result of QLT administration. More importantly, qPCR (Figure 4D), picrosirius red/Masson's trichrome staining (Figure 4E) and hydroxylproline quantification (Figure 4F) all suggested that ILK inhibition attenuated liver fibrosis. HSC proliferation (Figure 4G) and ROS production (Figure 4H) were down‐regulated by the ILK inhibitor. Western blotting confirmed that p38‐MAPK phosphorylation was weakened in the ILK inhibitor injected livers, compared to the control livers (Figure 4I). In an alternative therapeutic scenario in which QLT was given to the mice with developed liver fibrosis, it was discovered that ILK inhibition partially but effectively reversed liver fibrosis (Figure S4).

**FIGURE 4:**
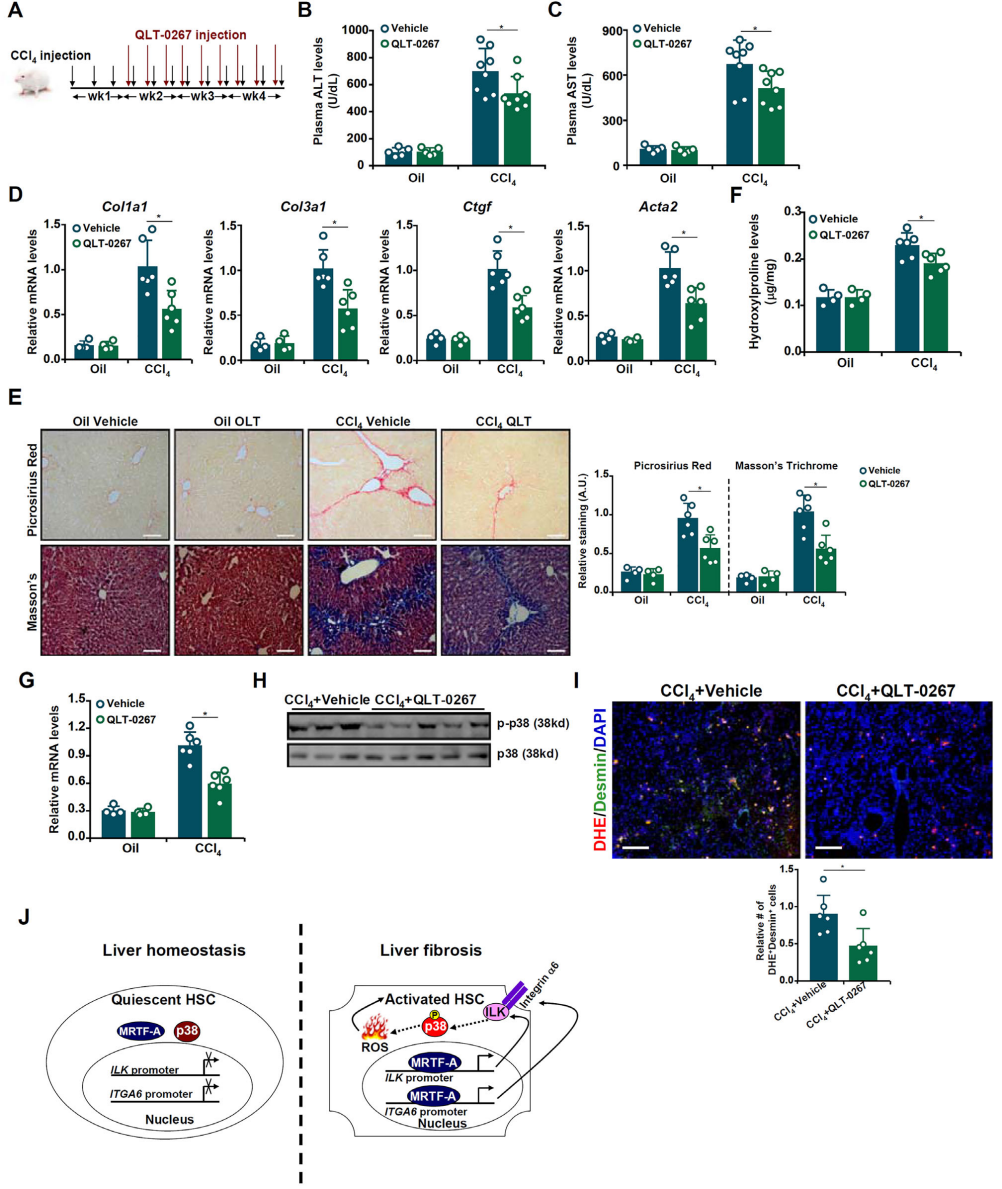
ILK inhibition attenuates liver fibrosis in mice. C57/BL6 mice were injected with CCl_4_ in the presence or absence of an ILK inhibitor (QLT‐0267, 10 mg/kg) for 4 weeks. (A) Scheme of animal protocol. (B) Plasma ALT levels. (C) Plasma AST levels. (D) Expression of pro‐fibrogenic genes was examined by qPCR. (E) Picrosirius red and Masson's trichrome staining. (F) Hydroxylproline levels. (G) Desmin expression was examined by qPCR. (H) Phosphorylation of p38‐MAPK was evaluated by western blotting. (I) Frozen sections were stained with DHE and an anti‐desmin antibody. (J) A schematic model. In quiescent HSCs, MRTF‐A is mostly cytoplasmic and *ITGA6*/*ILK* transcription is largely turned off. In trans‐differentiated HSCs, MRTF‐A trans‐locates into the nucleus and activates *ITGA6*/*ILK* transcription. Integrinα6‐ILK in turn activates p38‐MAPK signalling to promote ROS production and fuel HSC expansion leading to the pathogenesis of liver fibrosis

In summary, we describe a novel mechanism where nucleus‐initiated transcriptional activation of ITGA6/ILK by MRTF‐A serves to jumpstart cytoplasmic MAPK‐p38 signalling contributing to ROS‐fueled HSC proliferation (Figure 4J). Our data provide compelling evidence that MRTF‐A functions as a pivotal link between MAPK signalling, ROS production and myofibroblast (HSC) expansion in the pathogenesis of liver fibrosis. The novel mechanistic insights provided by this study may further incentivize the screening for small‐molecule compounds that target the MRTF‐A‐ITGA6/ILK‐MPAK axis to yield therapeutic solutions against liver fibrosis.

## CONFLICT OF INTEREST

The authors declare that they have no conflict of interest.

## Supporting information

Supporting Information

## References

[1] Mederacke I , Hsu CC , Troeger JS , et al. Fate tracing reveals hepatic stellate cells as dominant contributors to liver fibrosis independent of its aetiology. Nat. Commun. 2013;4:2823. 24264436 10.1038/ncomms3823 PMC4059406

[2] Small EM . The actin‐MRTF‐SRF gene regulatory axis and myofibroblast differentiation. J Cardiovasc Transl Res. 2012;5(6):794‐804. 22898751 10.1007/s12265-012-9397-0

[3] Wang DZ , Li S , Hockemeyer D , et al. Potentiation of serum response factor activity by a family of myocardin‐related transcription factors. Proc Natl Acad Sci U S A. 2002;99(23):14855‐14860. 12397177 10.1073/pnas.222561499 PMC137508

[4] Legate KR , Wickstrom SA , Fassler R . Genetic and cell biological analysis of integrin outside‐in signaling. Genes Dev. 2009;23(4):397‐418. 19240129 10.1101/gad.1758709

