# Supplementary material for: Myocardin‐related transcription factor A drives ROS‐fueled expansion of hepatic stellate cells by regulating p38‐MAPK signalling
Source: Clin Transl Med. 2022 Feb 20;12(2):e688. doi: 10.1002/ctm2.688 (PMC8858634; doi:10.1002/ctm2.688)
Supplement: Supplementary file 1 — Supporting Information [file CTM2-12-e688-s001.docx]

**Dong W et al: Myocardin-related transcription factor A drives ROS-fueled expansion of hepatic stellate cells by regulating p38-MAPK signaling**

**Online supplementary material**

**Supplementary Methods**

*Animals*

All animal protocols were reviewed and approved the intramural Ethics Committee on Humane Treatment of Laboratory Animals of Nanjing Medical University. The mice were maintained in an SPF environment with 12 h light/dark cycles and libitum access to food and water. Germline MRTF-A knockout mice have been described previously ([1-3](#_ENREF_1)). *Mrtfa*^f/f^ mice ([4](#_ENREF_4)) were crossed to *Postn*-Cre^ERT2^ mice ([5](#_ENREF_5)) to generate myofibroblast-specific MRTF-A knockout mice. Liver fibrosis was induced by CCl_4_ injection (1.0mL/kg as 50% vol/vol) as previously described ([6](#_ENREF_6), [7](#_ENREF_7)). Tamoxifen dissolved in peanut oil (100 mg/kg body weight) was given by i.p. injection for 7 consecutive days.

*Cell culture*

Human immortalized hepatic stellate cells (LX-2, ATCC) were maintained in DMEM supplemented with 10% FBS. Primary hepatic stellate cells were isolated and maintained as previously described ([8](#_ENREF_8)). Human *ITGA6* promoter-luciferase constructs ([9](#_ENREF_9)), *ILK* promoter-luciferase constructs ([10](#_ENREF_10)), and MRTF-A expression constructs ([11](#_ENREF_11), [12](#_ENREF_12)) have been previously described. Small interfering RNAs were purchased from Dharmacon: for human *ITGA6* siRNA, CCAUCACAGUAACUCCUAA; for human ILK siRNA, GTCAAGTTCTCTTTCCAAT; for mouse *Itga6* siRNA, GACCAAAGACTCGATGTTT; for mouse *Ilk* siRNA, GACGCTCAGCAGACATGTGGA. Transient transfections were performed with Lipofectamine 2000. Luciferase activities were assayed 24-48 hours after transfection using a luciferase reporter assay system (Promega) as previously described.

*Protein extraction and Western blot*

Whole cell lysates were obtained by re-suspending cell pellets in RIPA buffer (50 mM Tris pH7.4, 150 mM NaCl, 1% Triton X-100) with freshly added protease inhibitor (Roche) as previously described ([13-15](#_ENREF_13)). Western blot analyses were performed with anti-FLAG (Sigma, F3165), anti-p38 (Abcam, ab170099), anti-phosphorylated p38 (Cell Signaling Tech, 4511), anti-ERK (Cell Signaling Tech, 5013), anti-phosphorylated ERK (Cell Signaling Tech, 9101), anti-JNK (Proteintech, 24164-1), anti-phosphorylated ERK (Cell Signaling Tech, 9251), anti-integrin α6 (Proteintech, 27189-1), anti-ILK (Proteintech, 12955-1), and anti-β-actin (Sigma, A2228) antibodies.

*RNA Isolation and Real-time PCR*

RNA was extracted with the RNeasy RNA isolation kit (Qiagen). Reverse transcriptase reactions were performed using a SuperScript First-strand Synthesis System (Invitrogen) as previously described ([16](#_ENREF_16), [17](#_ENREF_17)). Real-time PCR reactions were performed on an ABI Prism 7500 system with the following primers: for human *ITGA6*, 5’- ATGCACGCGGATCGAGTTT-3’ and 5’-TTCCTGCTTCGTATTAACATGCT-3’; for human *ILK*, 5’-GACGACATTTTCACTCAGTGCC-3’ and 5’-ACGGTTCATTACATTGATCCGTG-3’; for mouse *Itga6*, 5’-TGCAGAGGGCGAACAGAAC-3’ and 5’-GCACACGTCACCACTTTGC-3’; for mouse *Ilk*, 5’-ACGACATTTTCACTCAGTGCC-3’ and 5’-TCATCCCCACGATTCATCACAT-3’; for mouse *Col1a1*, 5’-ATTTGAAGTCCCAGAAAG-3’ and 5’-AGAAACTCCCGTCTGCTC-3’; for mouse *Acta2*, 5’-CCTGTTTCGGGAGCAGAA-3’ and 5’-GGTTATATAGCCCCCTGG-3’; for mouse *Col3a1*, 5’-GACTCTGGCAAAACTCAAAGTATCA-3’ and 5’-TAGGAATGTGCTTTGTGATAGCCT-3’; for mouse *Lox*, 5’-ACGTTTCCAATCACATTACG-3’ and 5’-ACGGTCCTCCTCTCCCCTTT-3’; for mouse *Ctgf*, 5’-CTTCTGCGATTTCGGCTCC-3’ and 5’-TACACCGACCCACCGAAGA-3’; for mouse *Timp1*, 5’-CCAGAGCCGTCACTTTGCTT-3’ and 5’-AGGAAAAGTAGACAGTGTTCAGGCTT-3’. Ct values of target genes were normalized to the Ct values of housekeekping control gene (18s, 5’-CGCGGTTCTATTTTGTTGGT-3’ and 5’-TCGTCTTCGAAACTCCGACT-3’ for both human and mouse genes) using the ΔΔCt method and expressed as relative mRNA expression levels compared to the control group which is arbitrarily set as 1.

*DHE and DCFH-DA staining*

DHE and DCFH-DA stainings were performed essentially as previously described ([18](#_ENREF_18)). Frozen liver sections or cells were stained with DHE (10 μM) or DCFH-DA (10 μM) at 37°C for 30 min. Fluorescence was visualized by co-focal microscopy (LSM 710, Zeiss). Quantifications were performed with Image J. 3 slides were stained from each individual mouse and ~5 fields counted per slide.

*PCR array*

A customized PCR array (Qiagen) in a 96-well format was performed to screen for MRTF-A target genes. 1μg total RNA extracted from LX-2 cells was reverse-transcribed using the RT^2^ First Strand kit supplied by the vendor. Then, the cDNA was mixed with 2x RT^2^ SYBR Green Mastermix and 25μl of the mix was dispensed into the customized 96-well plate that contained 45 pre-selected genes in duplicate plus 3 housekeeping genes for normalization. Quantitative PCR was performed on an Applied Biosystems StepOne Plus system. Cycle threshold (CT) values were calculated using StepOne software v2.1. The fold-change for each gene was calculated using the ΔΔCT method and normalized by the housekeeping genes.

*Chromatin Immunoprecipitation (ChIP)*

Chromatin immunoprecipitation (ChIP) assays were performed essentially as described before ([19-22](#_ENREF_19)). In brief, chromatin in control and treated cells were cross-linked with 1% formaldehyde. Cells were incubated in lysis buffer (150 mM NaCl, 25 mM Tris pH 7.5, 1% Triton X-100, 0.1% SDS, 0.5% deoxycholate) supplemented with protease inhibitor tablet and PMSF. DNA was fragmented into ~200 bp pieces using a Branson 250 sonicator. Aliquots of lysates containing 200 μg of protein were used for each immunoprecipitation reaction with anti-FLAG or pre-immune IgG.

*Statistical analysis*

One-way ANOVA with post-hoc Scheff´e analyses were performed by SPSS software (IBM SPSS v18.0, Chicago, IL, USA). Unless otherwise specified, values of *p*＜0.05 were considered statistically significant.

**Supplementary Discussion**

Despite the advances brought out by the present report to the field of liver pathobiology, several lingering issues and limitations remain to be addressed. First, it is not entirely clear whether ROS, acting as a fuel for HSC proliferation, are derived from. During liver injury, ROS can be produced in hepatocytes ([23](#_ENREF_23), [24](#_ENREF_24)), sinusoidal endothelial cells ([25](#_ENREF_25)), Kupffer cells ([26](#_ENREF_26), [27](#_ENREF_27)), and HSCs ([18](#_ENREF_18), [28](#_ENREF_28)). Indeed, expression profiling indicates that NADPH oxidase (NOX) isoforms, which are major ROS producers, can be detected with varying abundance in different types of hepatic cells ([29](#_ENREF_29), [30](#_ENREF_30)). It is possible that ROS, originated from different cellular sources, converge on HSCs to drive proliferation. Second, the mechanistic link between ROS accumulation and MRTF-A activation is missing. One of the paradigms with regard to the regulation of MRTF-A activity is its cytoplasm-nucleus trans-location ([31](#_ENREF_31)). We have previously shown that MRTF-A nuclear accumulation can be stimulated by increased ROS levels (oxLDL) in vascular endothelial cells ([32](#_ENREF_32)). In addition, myocardial ischemia-reperfusion injury, via up-regulation of ROS production, can prompt MRTF-A nuclear shuttling in macrophages ([33](#_ENREF_33)). It is tempting to speculate that ROS might activate MRTF-A in HSCs by promoting its nuclear enrichment. Third, we focused on the regulation of integrin signaling by MRTF-A in the present study. It remains unknown this process, which appears to be critical for HSC activation, represents the predominant pathway influenced by MRTF-A deficiency. A genomewide transcriptomic analysis comparing the *MRTF-A*^f/f^ HSCs and *MRTF-A*^ΔMF^ HSCs would be of help to clarify this issue. Finally, the proposed model wherein MRTF-A regulates ROS-fueled HSC expansion by regulating ITGA6/ILK transcription needs to be validated in humans.

**References**

1. Fan Z, Li N, Xu Z, Wu J, Fan X, Xu Y. An interaction between MKL1, BRG1, and C/EBPbeta mediates palmitate induced CRP transcription in hepatocytes. Biochimica et biophysica acta Gene regulatory mechanisms. 2019;1862(9):194412. Epub 2019/07/30.

2. Lu Y, Lv F, Kong M, Chen X, Duan Y, Sun D, et al. A cAbl-MRTF-A Feedback Loop Contributes to Hepatic Stellate Cell Activation. Frontiers in cell and developmental biology. 2019;7:243. Epub 2019/11/05.

3. Yang Y, Yang G, Yu L, Lin L, Liu L, Fang M, et al. An Interplay Between MRTF-A and the Histone Acetyltransferase TIP60 Mediates Hypoxia-Reoxygenation Induced iNOS Transcription in Macrophages. Frontiers in cell and developmental biology. 2020;8:484. Epub 2020/07/07.

4. Li Z, Chen B, Dong W, Kong M, Fan Z, Yu L, et al. MKL1 promotes endothelial-to-mesenchymal transition and liver fibrosis by activating TWIST1 transcription. Cell death & disease. 2019;10(12):899. Epub 2019/11/30.

5. Kanisicak O, Khalil H, Ivey MJ, Karch J, Maliken BD, Correll RN, et al. Genetic lineage tracing defines myofibroblast origin and function in the injured heart. Nature communications. 2016;7:12260. Epub 2016/07/23.

6. Li Z, Lv F, Dai C, Wang Q, JIang C, Fang M, et al. Activation of galectin-3 (LGALS3) transcription by injurious stimuli in the liver is commonly mediated by BRG1. Frontiers in cell and developmental biology. 2019;7:310.

7. Dong W, Kong M, Zhu Y, Shao Y, Wu D, Lu J, et al. Activation of TWIST Transcription by Chromatin Remodeling Protein BRG1 Contributes to Liver Fibrosis in Mice. Frontiers in cell and developmental biology. 2020;8:340. Epub 2020/06/02.

8. Kong M, Hong W, Shao Y, Lv F, Fan Z, Li P, et al. Ablation of serum response factor in hepatic stellate cells attenuates liver fibrosis. J Mol Med (Berl). 2019;97(11):1521-33. Epub 2019/08/23.

9. Lin CS, Chen Y, Huynh T, Kramer R. Identification of the human alpha6 integrin gene promoter. DNA and cell biology. 1997;16(8):929-37. Epub 1997/08/01.

10. Melchior C, Kreis S, Janji B, Kieffer N. Promoter characterization and genomic organization of the gene encoding integrin-linked kinase 1. Biochimica et biophysica acta. 2002;1575(1-3):117-22. Epub 2002/05/22.

11. Yang Y, Li Z, Guo J, Xu Y. Deacetylation of MRTF-A by SIRT1 defies senescence induced down-regulation of collagen type I in fibroblast cells. Biochimica et biophysica acta Molecular basis of disease. 2020;1866(5):165723. Epub 2020/02/18.

12. Wu T, Wang H, Xin X, Yang J, Hou Y, Fang M, et al. An MRTF-A-Sp1-PDE5 Axis Mediates Angiotensin-II-Induced Cardiomyocyte Hypertrophy. Frontiers in cell and developmental biology. 2020;8:839. Epub 2020/10/06.

13. Sun L, Chen B, Wu J, Jiang C, Fan Z, Feng Y, et al. Epigenetic regulation of a disintegrin and metalloproteinase (ADAM) promotes colorectal cancer cell migration and invasion. Frontiers in cell and developmental biology. 2020(8):581692.

14. Mao L, Liu L, Zhang T, Qin H, Wu X, Xu Y. Histone Deacetylase 11 Contributes to Renal Fibrosis by Repressing KLF15 Transcription. Frontiers in cell and developmental biology. 2020;8:235. Epub 2020/05/05.

15. Yang Y, Liu L, Li M, Cheng X, Fang M, Zeng Q, et al. The chromatin remodeling protein BRG1 links ELOVL3 trans-activation to prostate cancer metastasis. Biochimica et biophysica acta Gene regulatory mechanisms. 2019;1862(8):834-45. Epub 2019/06/04.

16. Zhao Q, Yang J, Chen H, Li J, Que L, Zhu G, et al. Peli1 induction impairs cardiac microvascular endothelium through Hsp90 dissociation from IRE1alpha. Biochimica et biophysica acta Molecular basis of disease. 2019;1865(10):2606-17. Epub 2019/07/02.

17. Liu L, Mao L, Wu X, Wu T, Liu W, Yang Y, et al. BRG1 regulates endothelial-derived IL-33 to promote ischemia-reperfusion induced renal injury and fibrosis in mice. Biochimica et biophysica acta Molecular basis of disease. 2019;1865(9):2551-61. Epub 2019/06/23.

18. Kong M, Chen X, Lv F, Ren H, Fan Z, Qin H, et al. Serum response factor (SRF) promotes ROS generation and hepatic stellate cell activation by epigenetically stimulating NCF1/2 transcription. Redox biology. 2019;26:101302. Epub 2019/08/24.

19. Chen B, Zhao Q, Xu T, Yu L, Zhuo L, Yang Y, et al. BRG1 activates PR65A transcription to regulate NO bioavailability in vascular endothelial cell. Frontiers in cell and developmental biology. 2020;8:774.

20. Chen B, Yuan Y, Sun L, Chen J, Yang M, Yin Y, et al. MKL1 Mediates TGF-β Induced RhoJ Transcription to Promote Breast Cancer Cell Migration and Invasion. Frontiers in cell and developmental biology. 2020;8:832.

21. Chen B, Fan Z, Sun L, Chen J, Feng Y, Fan X, et al. Epigenetic activation of the small GTPase TCL contributes to colorectal cancer cell migration and invasion. Oncogenesis. 2020;9(9):86. Epub 2020/10/02.

22. Li Z, Xia J, Fang M, Xu Y. Epigenetic regulation of lung cancer cell proliferation and migration by the chromatin remodeling protein BRG1. Oncogenesis. 2019;8(11):66. Epub 2019/11/07.

23. Bhogal RH, Curbishley SM, Weston CJ, Adams DH, Afford SC. Reactive oxygen species mediate human hepatocyte injury during hypoxia/reoxygenation. Liver transplantation : official publication of the American Association for the Study of Liver Diseases and the International Liver Transplantation Society. 2010;16(11):1303-13. Epub 2010/10/30.

24. Li Z, Zhao Q, Lu Y, Zhang Y, Li L, Li M, et al. DDIT4 S-Nitrosylation Aids p38-MAPK Signaling Complex Assembly to Promote Hepatic Reactive Oxygen Species Production. Adv Sci (Weinh). 2021;8(18):2101957. Epub 2021/07/27.

25. Li Z, Chen B, Dong W, Kong M, Shao Y, Fan Z, et al. The chromatin remodeler Brg1 integrates ROS production and endothelial-mesenchymal transition to promote liver fibrosis in mice. Front Dev Cell Biol. 2019;7:245.

26. Kim SY, Jeong JM, Kim SJ, Seo W, Kim MH, Choi WM, et al. Pro-inflammatory hepatic macrophages generate ROS through NADPH oxidase 2 via endocytosis of monomeric TLR4-MD2 complex. Nature communications. 2017;8(1):2247. Epub 2017/12/23.

27. Huang H, Chen HW, Evankovich J, Yan W, Rosborough BR, Nace GW, et al. Histones activate the NLRP3 inflammasome in Kupffer cells during sterile inflammatory liver injury. J Immunol. 2013;191(5):2665-79. Epub 2013/08/02.

28. Gandhi CR. Oxidative Stress and Hepatic Stellate Cells: A PARADOXICAL RELATIONSHIP. Trends in cell & molecular biology. 2012;7:1-10. Epub 2012/01/01.

29. Crosas-Molist E, Fabregat I. Role of NADPH oxidases in the redox biology of liver fibrosis. Redox biology. 2015;6:106-11. Epub 2015/07/24.

30. Liang S, Kisseleva T, Brenner DA. The Role of NADPH Oxidases (NOXs) in Liver Fibrosis and the Activation of Myofibroblasts. Frontiers in physiology. 2016;7:17. Epub 2016/02/13.

31. Olson EN, Nordheim A. Linking actin dynamics and gene transcription to drive cellular motile functions. Nature reviews Molecular cell biology. 2010;11(5):353-65. Epub 2010/04/24.

32. Fang F, Yang Y, Yuan Z, Gao Y, Zhou J, Chen Q, et al. Myocardin-related transcription factor A mediates OxLDL-induced endothelial injury. Circulation research. 2011;108(7):797-807. Epub 2011/02/19.

33. Yu L, Yang G, Zhang X, Wang P, Weng X, Yang Y, et al. Megakaryocytic Leukemia 1 (MKL1) Bridges Epigenetic Activation of NADPH Oxidase in Macrophages to Cardiac Ischemia-Reperfusion Injury. Circulation. 2018;138(24):2820-36. Epub 2018/07/19.

**Fig.S1:** *MRTF-A*^f/f^ mice and *MRTF-A*^ΔMF^ were induced to develop liver fibrosis by CCl4 injection for 4 weeks. Tamoxifen (100mg/kg) was given by i.p. injection for 7 days. (A) Scheme of animal protocol. (**B**) MRTF-A expression in primary HSCs and primary hepatocytes were examined by Western blotting.

**Fig.S2:** PCR array was performed as described in Methods. (**A**) Raw PCR data. (**B**) Column graph. Note that dCt=-log_2_(KO/WT).

**Fig.S3:** (**A-C**) LX-2 cells were infected with lentivirus carrying MRTF-A CA or an empty vector (EV) followed by transfection with indicated siRNAs. ROS levels were evaluated by DHE staining. Proliferation was examined by EdU staining. MAPK phosphorylation was examined by Western. (**D-F**) Primary murine HSCs were infected with lentivirus carrying MRTF-A CA or an empty vector (EV) followed by transfection with indicated siRNAs. ROS levels were evaluated by DHE staining. Proliferation was examined by EdU staining. MAPK phosphorylation was examined by Western.

**Fig.S4:** C57/BL6 mice were injected with CCl_4_ in the presence or absence of an ILK inhibitor (QLT-0267, 10mg/kg) for 7wk. (**A**) Scheme of animal protocol. (**B**) Plasma ALT levels. (**C**) Plasma AST levels. (**D**) Expression of pro-fibrogenic genes were examined by qPCR. (**E**) Picrosirius red and Masson’s trichrome staining.
